# Virtual Reality-Based Orienteering Training Improves Spatial Memory in College Students with Operationally Defined DTD-like Navigational Difficulties: fNIRS Correlates of Training-Related Cortical Adaptation

**DOI:** 10.3390/jintelligence14080187

**Published:** 2026-08-16

**Authors:** Yang Liu, Heying Liu, Pengyang Kang

**Affiliations:** Key Laboratory of Sports Learning Science, Shaanxi Normal University, Xi’an 710119, China; liuyang0330@snnu.edu.cn

**Keywords:** developmental topographical disorientation, operationally defined DTD-like navigational difficulties, virtual reality, orienteering, spatial memory, neuroplasticity, fNIRS

## Abstract

Background: Developmental topographical disorientation (DTD)-like navigational difficulties can impair everyday orientation and spatial information processing. Objective: We examined whether 8-week virtual reality (VR) orienteering training improves performance on a laboratory spatial memory task in college students with operationally defined DTD-like navigational difficulties and examined task-related cortical changes. Methods: In a randomized controlled trial, 96 students were assigned to VR orienteering, VR exercise, or control groups (*n* = 32/group). The exercise groups trained twice weekly for 45 min for 8 weeks at moderate intensity (64–76% HRmax). Before and after the intervention, all participants completed a delayed visuospatial recognition task designed to assess spatial-memory-related processing, while functional near-infrared spectroscopy measured hemodynamic changes in prefrontal and sensorimotor cortices. The post-test session was conducted 48 h after the final scheduled session, with no immediate post-exercise assessment. Results: VR orienteering produced greater improvement in spatial-memory-task accuracy and reaction time than the other groups. After BH-FDR correction across eight ROIs, training-specific reductions in task-related activation were observed in the right primary motor cortex and left frontopolar area. Within the VR orienteering group, pre-to-post change in left orbitofrontal activation was negatively associated with change in spatial-memory-task accuracy, Pearson *r*(30) = −0.819, 95% CI [−0.908, −0.658], *p* < 0.001, *R*^2^ = 0.670; the association remained significant after eight-ROI BH-FDR correction (adjusted *p* < 0.001). Conclusions: VR orienteering improved performance on the laboratory spatial memory task and was accompanied by reduced task-related cortical recruitment. Greater reductions in L-OFC activation were linked to greater improvements in accuracy, identifying L-OFC activation change as a neural correlate of training-related spatial-memory improvement.

## 1. Introduction

Developmental Topographical Disorientation (DTD) refers to a severe and persistent impairment in spatial navigation that begins early in life and occurs in the absence of acquired brain injury or major intellectual impairment (Iaria et al., 2009, 2014; Kim et al., 2015). DTD-like navigational difficulties can involve getting lost in familiar environments and difficulty forming effective environmental representations (Iaria et al., 2009, 2014; Kim et al., 2015). In this study, the target group was defined operationally through screening and interview criteria, allowing navigation-related difficulties to be examined in a college sample. Spatial memory, understood here as the encoding, maintenance, updating, and retrieval of spatial relations and visual-spatial layouts, provides an important cognitive foundation for orientation and environmental information use (Ishikawa & Montello, 2006; Lubinski, 2010; van der Ham et al., 2020; Weisberg et al., 2019). Recent work has emphasized combining self-report screening with direct navigational performance when characterizing DTD-like populations (Bonavita et al., 2025). The present study focused on the laboratory spatial-memory component of this broader navigation profile.

Physical exercise, particularly complex exercise involving cognitive participation, has been associated with benefits for cognitive function (Bao et al., 2022; Erickson et al., 2011; Singh et al., 2025; Stillman et al., 2020). Orienteering is an open-skill activity that combines physical movement with map reading, route planning, and environmental matching (Eccles et al., 2002; Stroth et al., 2009). These demands require continuous updating of spatial information during movement and make orienteering a natural training context for spatial-memory-related learning. The present study isolated this cognitive component in a standardized laboratory task, while VR provided the control and consistency needed for repeated training and multimodal data acquisition.

The maturation of virtual reality (VR) technology provides a controllable platform for addressing these challenges. VR enables the construction of standardized simulated environments while retaining multisensory spatial information (Bohil et al., 2011; Llorens et al., 2026; Mouatt et al., 2020). Navigation training in VR has been reported to influence spatial-memory-related and wayfinding measures (Llorens et al., 2026). Functional near-infrared spectroscopy (fNIRS), with its portability and relative tolerance of movement, can monitor cortical hemodynamic responses during naturalistic motor–cognitive tasks and is therefore useful for examining accessible prefrontal and sensorimotor regions (Herold et al., 2018).

At the neural-mechanism level, spatial representation encoding, maintenance, updating, retrieval, and strategy selection depend on distributed prefrontal, frontoparietal, hippocampal, retrosplenial, and sensorimotor systems (Bhattacharjee et al., 2021; Marek & Dosenbach, 2018; Murphy et al., 2020). DTD-specific neuroimaging evidence converges on altered recruitment and communication within this distributed network. Initial case and group studies implicated atypical hippocampal, retrosplenial, and hippocampal-prefrontal responses during spatial processing (Iaria et al., 2009, 2014; Kim et al., 2015). Further single-case studies reported absent task-related activation in route-knowledge regions and reduced structural integrity of parieto-prefrontal and parieto-premotor pathways (Palermo et al., 2014; Rusconi et al., 2021). Collectively, these findings frame DTD-related disorientation as a network-level disturbance in spatial representation. A 2024 group-level network analysis also suggests compensatory organization (Faryadras et al., 2024), indicating that focal under-recruitment and compensatory network efficiency can coexist. The latest systematic review emphasized poor mental-map quality as a central behavioral characteristic while noting heterogeneity in definitions and assessment (van der Ham & Claessen, 2026).

Within the accessible cortical network, the frontopolar area (FPA) contributes to higher-order cognitive control and information integration, whereas the primary motor cortex (M1) contributes to sensorimotor integration and bodily representations of action (Bhattacharjee et al., 2021; Marek & Dosenbach, 2018; Murphy et al., 2020). The neural-efficiency hypothesis proposes that better performance can sometimes co-occur with lower task-related activation, with the meaning of activation changes shaped by task demands and individual differences (Neubauer & Fink, 2009; Poldrack et al., 2005). These ideas frame the present fNIRS approach: accessible cortical responses can reveal how training reshapes the cortical support of spatial memory, while hippocampal and retrosplenial components remain outside the measurement. Prior studies linking navigation experience to hippocampal structure and aerobic training to hippocampal volume and memory used different populations, tasks, and imaging methods (Erickson et al., 2011; Maguire et al., 2000); they provide a broader context for the present cortical findings. We therefore expected repeated VR orienteering to strengthen executive control and sensorimotor integration, accompanied by changes in prefrontal and sensorimotor responses during the spatial memory task (Wang et al., 2026).

In summary, this study uses VR-based orienteering as an intervention for individuals with operationally defined DTD-like navigational difficulties. Combining a randomized controlled design with behavioral measurements and fNIRS, we examined three linked questions: (1) whether 8 weeks of VR-based orienteering training improves performance on a laboratory spatial memory task; (2) whether behavioral improvement is accompanied by reduced task-related activation in accessible prefrontal-sensorimotor cortex; and (3) whether pre-to-post changes in L-OFC activation are associated with pre-to-post changes in spatial-memory-task accuracy in the VR orienteering group. Together, these questions connect the behavioral effect of training with its cortical signature.

Based on the neural-efficiency hypothesis and embodied-cognition theory, three hypotheses were proposed. First, the VR orienteering group was expected to show greater improvement in spatial-memory-task accuracy and reaction time than the VR exercise and control groups after the 8-week intervention. Second, VR orienteering was expected to be accompanied by reduced task-related cortical activation within the prefrontal-sensorimotor network, a pattern consistent with more efficient neural recruitment. Third, in the VR orienteering group, the pre-to-post change in L-OFC task-related activation was expected to covary with the pre-to-post change in spatial-memory-task accuracy.

## 2. Subjects and Methods

### 2.1. Study Subjects

This study recruited college students from universities in Xi’an. A priori sample size estimation was performed using G*Power 3.1.9.2 software. Based on effect sizes reported in related young-adult exercise-cognition fNIRS studies (Li et al., 2024; Yin et al., 2024), the planning effect size was set at *f* = 0.35, α = 0.05, and statistical power (1 − *β*) = 0.80. The calculation indicated that a minimum of 28 participants per group was required, with a total sample size of at least 84. Accounting for potential participant dropout during the experiment, a total of 96 college students meeting the operational criteria for DTD-like navigational difficulties described below were ultimately recruited and randomly assigned using a random number table method into three groups: VR Orienteering Group, VR Exercise Group, and Control Group, with 32 participants in each group.

Operational Definition of DTD-Like Navigational Difficulties for This Study: Given that a strict clinical diagnosis of Developmental Topographical Disorientation (DTD) requires extensive neuropsychological testing that is not feasible in large-scale intervention studies, we adopted a well-established operational definition. All participants first completed the Familiarity and Spatial Cognitive Style Scale (FSCS), and those scoring below the 15th percentile were selected. Subsequently, a structured interview was conducted to confirm that participants consistently reported the core behavioral manifestations of DTD as proposed by Iaria et al. (2009): (1) getting lost frequently in very familiar surroundings; (2) experiencing orientation difficulties since childhood or adolescence; (3) having no other cognitive complaints; and (4) having no history of brain injury or neurological conditions. Thus, the participants in this study should be understood as a subclinical group with DTD-like navigational difficulties based on an operational definition. To avoid overdiagnosis, the term “operationally defined DTD-like navigational difficulties” is used throughout the manuscript.

Inclusion Criteria: (1) Meeting the operational criteria for DTD-like navigational difficulties described above (FSCS score below the 15th percentile and self-reported core symptoms); (2) Normal or corrected-to-normal vision, no color blindness or color weakness; (3) Right-handed; (4) No prior experience or training in orienteering; (5) No regular physical exercise habit in the past 6 months (moderate-intensity exercise less than 3 times per week, less than 30 min each time); (6) No 3D motion sickness (cybersickness) or related contraindications; (7) Familiar with computer keyboard operation and no prior participation in similar spatial cognition experiments; (8) Body Mass Index (BMI) within the normal range (18.5–23.9 kg/m^2^).

Exclusion Criteria: (1) Neurological disorders such as vascular dementia, Parkinson’s disease, epilepsy, or history of traumatic brain injury; (2) Psychiatric disorders such as severe depression or schizophrenia; (3) Severe systemic diseases involving the heart, liver, kidneys, or other health conditions unsuitable for moderate-intensity exercise; (4) Habitual use of cardinal directions (north, south, east, west) for daily orientation or giving directions.

All participants provided written informed consent before the experiment and committed to refraining from other motor skill learning or additional regular physical exercise during the experimental period. The study protocol was approved by the Academic Committee of Shaanxi Normal University (Approval No. 2024-193; Approval Date: 26 August 2024). No significant differences were found among the three groups in terms of sex distribution, age, BMI, or resting heart rate (all *p* > 0.05), indicating balanced group assignment and comparable baseline levels (see Table 1).

### 2.2. Experimental Design

A 3 (Group: VR Orienteering Group, VR Exercise Group, Control Group) × 2 (Test Time: pre-intervention, post-intervention) two-factor mixed experimental design was adopted. Group was a between-subjects variable, and Test Time was a within-subjects variable. The behavioral outcomes were accuracy (ACC) and reaction time (RT) on the spatial memory task. Neural outcomes were task-related beta coefficients derived from functional near-infrared spectroscopy (fNIRS) in eight prefrontal-sensorimotor ROIs. The post-test session, including the spatial memory task and concurrent task-related fNIRS acquisition, was conducted 48 h after the final scheduled session; no outcome assessment was performed immediately after training.

### 2.3. Experimental Procedure

**Figure 1:**
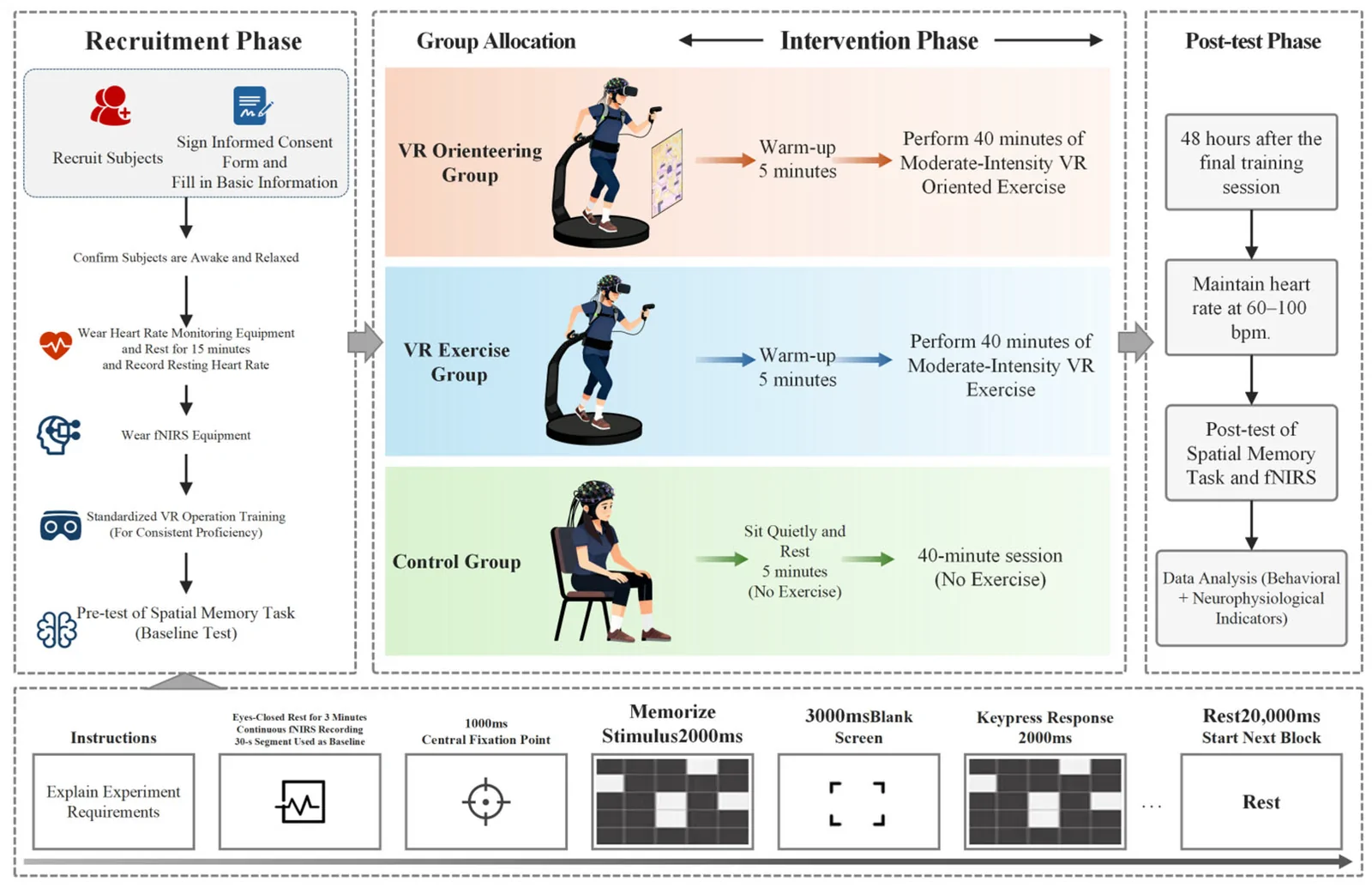
Schematic diagram of the experimental procedure. The post-test session, including the Spatial Memory Task and concurrent task-related fNIRS acquisition, was conducted 48 h after the final scheduled session; no outcome assessment was performed immediately after training.

The experimental procedure consisted of a practice phase, pre-test, 8-week intervention, and post-test. The post-test session, including the spatial memory task and concurrent task-related fNIRS acquisition, was conducted 48 h after the final scheduled session; no outcome assessment was performed immediately after training (Figure 1).

(1) Practice Phase: The experimenter explained the operation method of the VR equipment and guided participants through hands-on practice to ensure consistent operational proficiency.
(2) Pre-test: Upon arriving at the laboratory, participants first received an explanation of the procedure and signed the informed consent form. They then completed basic information forms, wore a DOMOR sports watch to monitor heart rate, and sat quietly for 15 min before resting heart rate was recorded. Subsequently, they wore the fNIRS device and performed the spatial memory task, during which task-related brain activity data were collected.
(3) 8-week Intervention:

Intervention duration: 8 weeks, 2 sessions per week, 45 min per session.

Exercise intensity control: During the intervention, exercise intensity for both the VR Orienteering Group and the VR Exercise Group was controlled at a moderate level (64–76% of HRmax; McMorris & Hale, 2012). Individual maximal heart rate was calculated using the formula “208 − 0.7 × age,” and heart rate was monitored in real time using a DOMOR sports watch. Participants were instructed via voice prompts to adjust their pace to maintain the target intensity range. Group tasks: VR Orienteering Group: Participants independently planned routes and navigated to target points in a VR environment by reading maps (running-driven). VR Exercise Group: Participants traveled along a preset navigation route in the same environment (running-driven, without cognitive planning). Control Group: Participants remained seated quietly for 45 min per session, twice weekly for 8 weeks, without engaging in physical exercise.

The post-test session, including the spatial memory task and concurrent task-related fNIRS acquisition, was conducted 48 h after the final scheduled session; no outcome assessment was performed immediately after training. At the post-test visit, resting heart rate was confirmed within 60–100 bpm before data acquisition began.

### 2.4. Measurement Tools and Tasks

#### 2.4.1. Spatial Memory Task

The spatial memory task assessed delayed visuospatial maintenance, comparison, recognition, and decision making. It was designed as a visuospatial delayed-recognition paradigm: participants encoded the locations of white squares within a matrix, maintained the spatial configuration across a delay, and judged whether a later probe matched the remembered configuration. Unlike a canonical mental rotation test, which requires the angular transformation of an object (Vandenberg & Kuse, 1978), the present task required the delayed maintenance and recognition of a spatial configuration. This distinction is consistent with work that explicitly separates delayed recognition (maintenance) from mental rotation (manipulation) (Tang et al., 2020). In the broader context of orienteering, fNIRS evidence indicates that prefrontal dynamics vary with the interaction between orienteering experience and cognitive performance (Liu et al., 2024). The task therefore provides a focused laboratory measure of spatial-memory-related processing, centered on delayed maintenance and recognition; route memory, route learning, cognitive-map construction, and real-world navigation involve additional processes beyond this paradigm. The task was programmed in E-Prime 2.0.

Stimuli and Procedure: Each trial began with a red “+” fixation point presented for 1000 ms, followed by a memory stimulus comprising a spatial configuration of white squares within a matrix presented for 2000 ms, then a 3000 ms blank screen, and finally a probe stimulus presented for 2000 ms. Participants were required to judge whether the probe configuration matched the remembered configuration. They pressed the F key for “same” and the J key for “different.”

Block Design: The formal test consisted of 6 blocks, each containing 4 trials presented in random order, with a 20-s rest interval between blocks. A practice session (with trial-by-trial feedback) was conducted before the formal test to ensure understanding of the task. Before the formal test began, participants rested with their eyes closed for 3 min while fNIRS signals were recorded continuously; a 30-s segment of the resting-state recording was used as the baseline. The entire task lasted approximately 15 min.

Recorded Indicators: Accuracy (ACC), reaction time (RT), and concurrent fNIRS brain activation data.

#### 2.4.2. Virtual Reality Intervention System

In this study, a highly realistic VR orienteering system was independently developed using the Unity engine. The VR hardware consisted of an HTC VIVE Pro head-mounted display, two base stations, and two VIVE Trackers (HTC Corporation, New Taipei City, Taiwan). The head-mounted display had a resolution of 2880 × 1600 pixels, a maximum field of view of 120°, and a refresh rate of 90 Hz. The core design framework of this system was based on the spatial cognitive development theory proposed by Siegel and White (1975), aiming to guide users through a complete cognitive progression from landmark knowledge, to route knowledge, and finally to survey knowledge. The system constructed two types of typical virtual environments (campus and park) and preset a total of 32 exercise routes. It should be noted that the campus and park virtual environments used in this study were standardized scenarios constructed based on typical layouts, rather than simulations of any specific real campus familiar to the participants. This design ensured ecological validity of the experimental environment while fundamentally controlling for confounding variables arising from individual differences in prior familiarity with specific real-world environments, thereby ensuring that the observed effects originated from the intervention itself rather than from prior experience.

In terms of movement interaction, the system employed Natural Locomotion technology to achieve 1:1 realistic motion mapping using two VIVE Trackers attached to the participants’ feet and in-place stepping movements. To ensure the accuracy of displacement mapping, personalized calibration was performed for each participant before the experiment: participants were asked to step in place with a natural gait for 10 s, and the system recorded their average stride length as the baseline for displacement stride length in the virtual environment, thus achieving individualized 1:1 spatial displacement calibration. This setup effectively enhanced immersion and movement realism.

Two key navigation modes were set in the system to enable experimental manipulation: In the VR orienteering scenario, participants used an interactive 3D map and a virtual compass to independently plan routes and navigate to target points, a design aimed at promoting active construction of spatial knowledge. In the VR exercise control scenario, participants followed a preset blue ground navigation line and green directional arrows provided by the system in real time within the identical environment and route, without the need for independent planning, merely moving according to the instructions. The two scenarios were identical in visual environment, movement route, and exercise intensity, differing only in navigation mode, thereby effectively isolating the core cognitive variable of higher-order spatial planning. The VR Orienteering Group used the VR orienteering scenario, while the VR Exercise Group used the VR exercise control scenario.

### 2.5. Data Collection and Processing

#### 2.5.1. fNIRS Data Collection

A LIGHTNIRS portable functional near-infrared spectroscopy system (Shimadzu Corporation, Kyoto, Japan) was used. Light source wavelengths: 780, 805, 830 nm; sampling rate: 13.33 Hz. Optode arrangement: 2 (rows) × 8 (columns) array, with 8 emitters and 8 detectors, forming 20 effective measurement channels, with an inter-optode distance of 3.0 cm.

**Figure 2:**
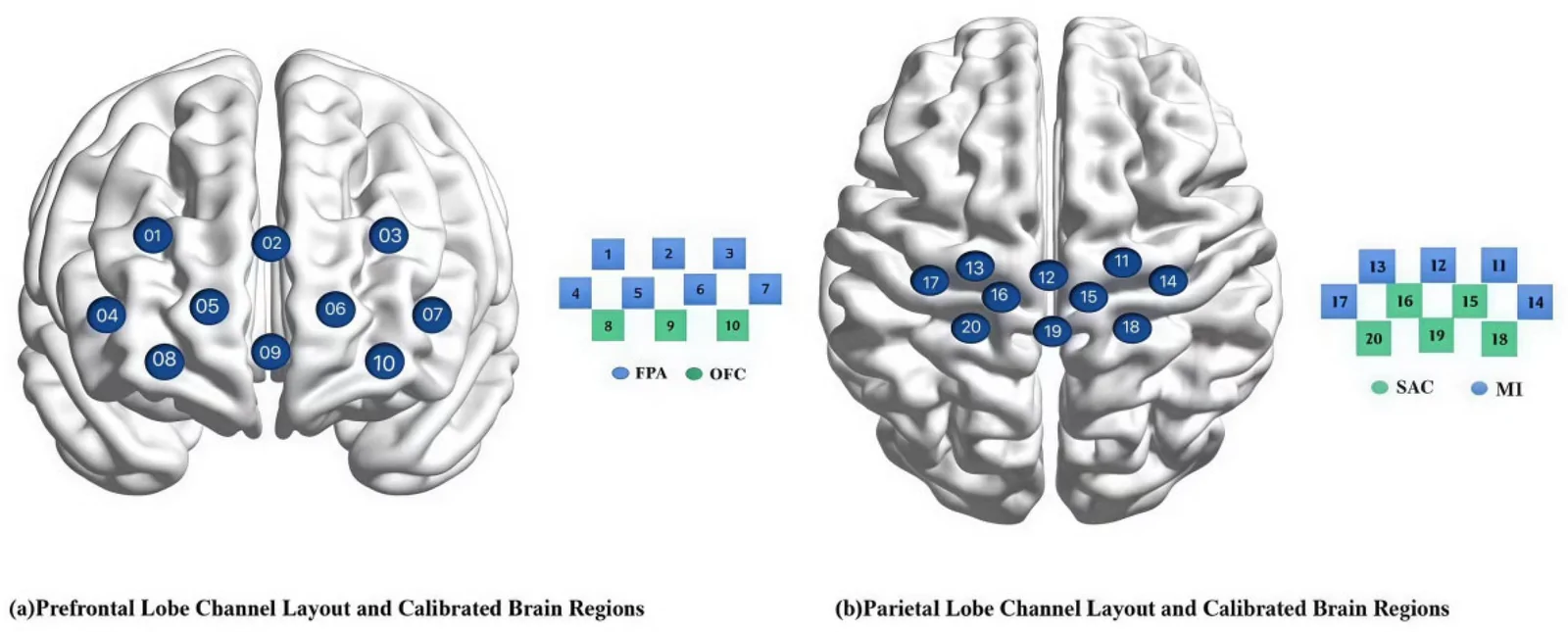
fNIRS channel layout and calibration brain region information.

A FASTRAK electromagnetic tracking system (3D Digitizer; Polhemus, Colchester, Vermont, USA) was used to acquire three-dimensional coordinates of the optodes, which were then mapped to MNI standard brain space using NIRS_SPM spatial probabilistic registration. Referring to Brodmann areas, eight regions of interest (ROIs) and their corresponding channels were identified (see Table 2, Figure 2). Some channels (e.g., channel 9) were located at the junction of functional brain areas; based on the spatial probabilistic registration results, they were simultaneously included in the analysis of adjacent ROIs, a common practice in fNIRS studies to capture activation signals from boundary regions.

#### 2.5.2. Data Processing and Statistical Analysis

Behavioral data (accuracy and reaction time on the spatial memory task) were analyzed using SPSS Statistics 25.0 software. A 3 (Group: VR Orienteering Group, VR Exercise Group, Control Group) × 2 (Time: pre-test, post-test) mixed-design analysis of variance (ANOVA) examined the main effects of Group and Time and their interaction. Significant interactions were followed by simple-effects analyses. Within each follow-up family, the three between-group contrasts at a given time point and the three within-group pre-post contrasts were Bonferroni corrected. All tests were two-tailed with α = 0.05. Omnibus ANOVAs are reported with F, numerator and denominator degrees of freedom, *p*, and partial eta squared (η_p_^2^). Paired contrasts are reported with t, df, Bonferroni-adjusted *p*, and Cohen’s dz; independent contrasts are reported with t, df, Bonferroni-adjusted *p*, and Hedges’ g.

fNIRS data were preprocessed and statistically analyzed using the NIRS-SPM toolbox on the MATLAB R2013b platform. Raw light-intensity signals were converted to optical density, and relative changes in oxygenated hemoglobin concentration were calculated using the modified Beer-Lambert law. Bandpass filtering (0.01–0.20 Hz) was applied to suppress low-frequency drift and high-frequency physiological noise, and motion artifacts were identified and corrected using a discrete wavelet transform (wavelet basis: ‘db4’, decomposition level: 5). Within the general linear model, the hemodynamic response function was convolved with the experimental design matrix and beta coefficients were extracted for each channel. For every participant and time point, channel beta coefficients were arithmetically averaged within each ROI; all fNIRS inferential analyses were conducted on these participant-level ROI means rather than on individual channels.

Multiplicity was controlled at the ROI level. For the mixed ANOVAs, Benjamini–Hochberg false discovery rate (BH-FDR) correction was applied separately across the eight ROI *p* values for the Group, Time, and Group × Time effects. Group × Time interactions that remained significant after this correction were followed by simple-effects analyses. To examine the post-intervention group differences, separate one-way group ANOVAs were conducted on the post-intervention values for each ROI, and BH-FDR correction was applied across the eight ROI omnibus *p* values. For each ROI that survived this correction, the three post-intervention pairwise group comparisons were Bonferroni corrected. For the brain-behavior analysis, change scores were calculated as pre-intervention minus post-intervention for each participant-level ROI mean and for spatial-memory accuracy and reaction time. Pearson correlations were calculated for all eight ROIs in each of the three groups. BH-FDR correction was applied across the eight ROI correlations separately within each Group × Outcome family. The focal brain-behavior analysis examined the relationship between L-OFC activation change and Spatial Memory Task accuracy change in the VR orienteering group. All *p* values are accompanied by the corresponding statistic and degrees of freedom. Partial eta squared (η_p_^2^), Cohen’s dz, Hedges’ g, and Pearson r were used as effect sizes for omnibus ANOVAs, paired contrasts, independent contrasts, and correlations, respectively; *R*^2^ is additionally reported for the focal correlation.

All behavioral and fNIRS records underwent data-quality screening before the brain-behavior analysis. Potentially influential observations were examined using studentized residuals, leverage, Cook’s distance, and leave-one-out estimates. All observations met the signal-quality and artifact criteria, and no recording, preprocessing, or transcription error was identified; no observation was removed. Each eight-ROI Pearson change-score family was multiplicity controlled using BH-FDR. Leave-one-out estimates and Spearman correlations using the same change scores were also calculated to examine whether the direction of the focal relationship was stable across analytic choices.

## 3. Results

### 3.1. Spatial Memory Task Performance Following VR Orienteering Training

To test Hypothesis 1, we examined whether VR orienteering produced greater changes in spatial-memory-task accuracy and reaction time than VR exercise and control. A 3 (Group: VR Orienteering Group, VR Exercise Group, Control Group) × 2 (Time: pre-intervention, post-intervention) mixed-design ANOVA was conducted for accuracy (ACC) and reaction time (RT). Descriptive and omnibus results are presented in Table 3; all simple-effect *p* values reported below are Bonferroni-adjusted.

Accuracy on the spatial memory task showed significant effects of Group, *F*(2, 93) = 13.008, *p* < 0.001, η_p_^2^ = 0.219, and Time, *F*(1, 93) = 107.925, *p* < 0.001, η_p_^2^ = 0.537, together with a significant Group × Time interaction, *F*(2, 93) = 34.163, *p* < 0.001, η_p_^2^ = 0.424. Baseline accuracy did not differ among groups, *F*(2, 93) = 0.051, *p* = 0.950, η_p_^2^ = 0.001. At post-intervention, accuracy was higher in the VR orienteering group than in the VR exercise group, *t*(93) = 5.193, Bonferroni-adjusted *p* < 0.001, Hedges’ g = 1.256, and in the control group, *t*(93) = 9.101, Bonferroni-adjusted *p* < 0.001, Hedges’ g = 2.201. The VR exercise group also exceeded the control group, *t*(93) = 3.907, Bonferroni-adjusted *p* < 0.001, Hedges’ g = 1.009. Within-group contrasts showed increased accuracy in the VR orienteering group, *t*(31) = 10.895, Bonferroni-adjusted *p* < 0.001, Cohen’s dz = 1.926, and VR exercise group, *t*(31) = 7.407, Bonferroni-adjusted *p* < 0.001, Cohen’s dz = 1.309, but not in the control group, *t*(31) = 0.335, Bonferroni-adjusted *p* = 1.000, Cohen’s dz = 0.059.

**Figure 3:**
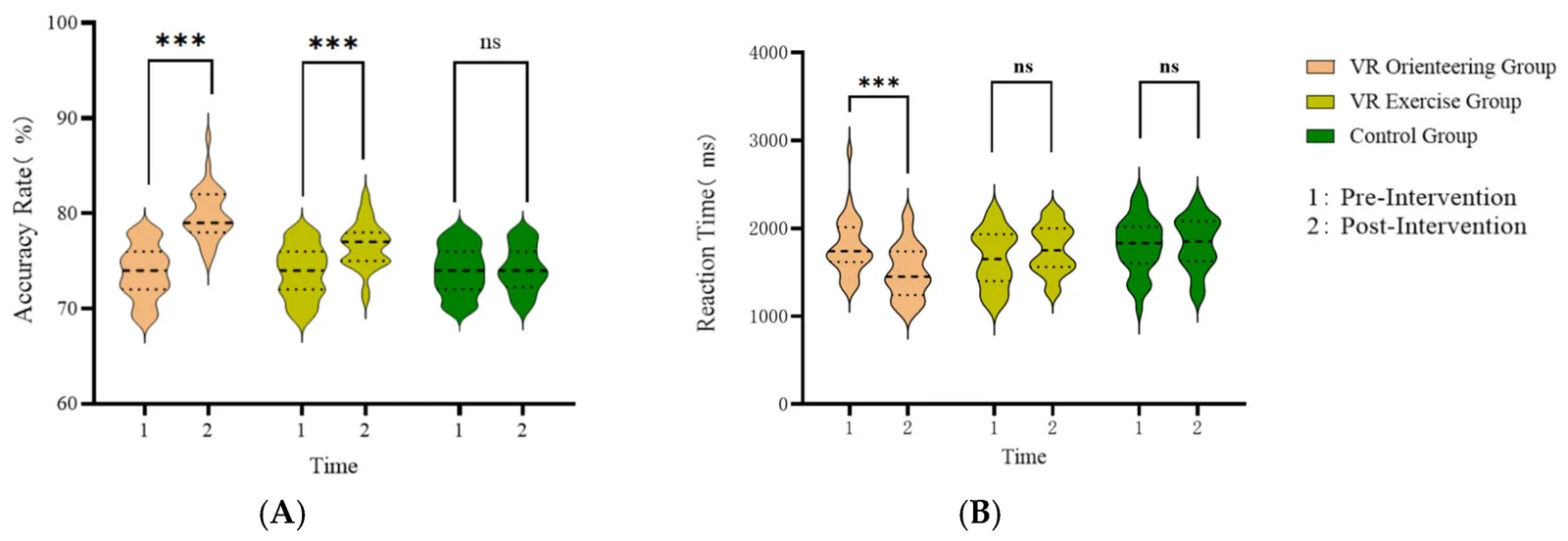
Changes in (**A**) spatial-memory-task accuracy and (**B**) reaction time before and after intervention across the three groups. The dashed line indicates the median, and the dotted lines indicate the first and third quartiles. *** *p* < 0.001; ns, not significant.

Reaction time on the spatial memory task showed no significant main effect of Group, *F*(2, 93) = 2.883, *p* = 0.061, η_p_^2^ = 0.058, or Time, *F*(1, 93) = 3.907, *p* = 0.051, η_p_^2^ = 0.040, but the Group × Time interaction was significant, *F*(2, 93) = 19.534, *p* < 0.001, η_p_^2^ = 0.296. At post-intervention, reaction time was shorter in the VR orienteering group than in the VR exercise group, *t*(93) = −3.506, Bonferroni-adjusted *p* = 0.002, Hedges’ g = −0.877, and control group, *t*(93) = −4.332, Bonferroni-adjusted *p* < 0.001, Hedges’ g = −1.035; the VR exercise and control groups did not differ, *t*(93) = −0.826, Bonferroni-adjusted *p* = 1.000, Hedges’ g = −0.209. Within-group contrasts showed a reduction only in the VR orienteering group, *t*(31) = −5.526, Bonferroni-adjusted *p* < 0.001, Cohen’s dz = −0.977. Changes in the VR exercise group, *t*(31) = 2.118, Bonferroni-adjusted *p* = 0.127, Cohen’s dz = 0.374, and control group, *t*(31) = 0.503, Bonferroni-adjusted *p* = 1.000, Cohen’s dz = 0.089, were not significant. Figure 3 shows the behavioral trends.

### 3.2. Task-Related Activation in Prefrontal–Sensorimotor Regions

To test Hypothesis 2, we examined whether VR orienteering was accompanied by reduced task-related cortical activation within the prefrontal-sensorimotor network. A 3 (Group: VR Orienteering Group, VR Exercise Group, Control Group) × 2 (Time: pre-intervention, post-intervention) mixed-design ANOVA was conducted for the participant-level mean beta coefficient of each ROI. BH-FDR correction was applied separately across the eight ROIs for each omnibus effect. Descriptive and corrected inferential results are presented in Table 4. Training-specific Group × Time effects remained significant only for R-M1 and L-FPA.

For R-M1, the Time effect, *F*(1, 93) = 14.739, BH-FDR-adjusted *p* < 0.001, η_p_^2^ = 0.137, and Group × Time interaction, *F*(2, 93) = 11.435, BH-FDR-adjusted *p* < 0.001, η_p_^2^ = 0.197, remained significant; the Group effect did not, *F*(2, 93) = 2.846, BH-FDR-adjusted *p* = 0.168, η_p_^2^ = 0.058. At post-intervention, R-M1 activation was lower in the VR orienteering group than in the VR exercise group, *t*(93) = −3.463, Bonferroni-adjusted *p* = 0.002, Hedges’ g = −0.846, and control group, *t*(93) = −5.008, Bonferroni-adjusted *p* < 0.001, Hedges’ g = −1.356; VR exercise and control did not differ, *t*(93) = −1.545, Bonferroni-adjusted *p* = 0.377, Hedges’ g = −0.356. Only the VR orienteering group showed a significant pre-post reduction, *t*(31) = −5.405, Bonferroni-adjusted *p* < 0.001, Cohen’s dz = −0.956; the VR exercise, *t*(31) = −1.595, adjusted *p* = 0.363, Cohen’s dz = −0.282, and control groups, *t*(31) = 1.133, adjusted *p* = 0.797, Cohen’s dz = 0.200, did not.

For L-FPA, the Time effect, *F*(1, 93) = 32.813, BH-FDR-adjusted *p* < 0.001, η_p_^2^ = 0.261, Group effect, *F*(2, 93) = 7.392, BH-FDR-adjusted *p* = 0.004, η_p_^2^ = 0.137, and Group × Time interaction, *F*(2, 93) = 8.791, BH-FDR-adjusted *p* = 0.001, η_p_^2^ = 0.159, remained significant. At post-intervention, activation was lower than control in both the VR orienteering group, *t*(93) = −4.725, Bonferroni-adjusted *p* < 0.001, Hedges’ g = −1.081, and VR exercise group, *t*(93) = −4.055, Bonferroni-adjusted *p* < 0.001, Hedges’ g = −0.955; the two VR groups did not differ, *t*(93) = −0.671, Bonferroni-adjusted *p* = 1.000, Hedges’ g = −0.193. Activation decreased from pre- to post-intervention in the VR orienteering group, *t*(31) = −5.503, Bonferroni-adjusted *p* < 0.001, Cohen’s dz = −0.973, and VR exercise group, *t*(31) = −4.131, Bonferroni-adjusted *p* < 0.001, Cohen’s dz = −0.730, but not in the control group, *t*(31) = −0.030, Bonferroni-adjusted *p* = 1.000, Cohen’s dz = −0.005.

For R-FPA, neither the Time effect, *F*(1, 93) = 3.331, BH-FDR-adjusted *p* = 0.114, η_p_^2^ = 0.035, the Group effect, *F*(2, 93) = 1.152, BH-FDR-adjusted *p* = 0.622, η_p_^2^ = 0.024, nor the Group × Time interaction, *F*(2, 93) = 0.755, BH-FDR-adjusted *p* = 0.540, η_p_^2^ = 0.016, was significant after correction. Accordingly, R-FPA was not interpreted as showing a training-specific neural change.

For the remaining ROIs, no Group × Time interaction survived correction (Table 4). R-SAC showed corrected main effects of Time, *F*(1, 93) = 9.487, BH-FDR-adjusted *p* = 0.007, η_p_^2^ = 0.093, and Group, *F*(2, 93) = 8.013, BH-FDR-adjusted *p* = 0.004, η_p_^2^ = 0.147, but no corrected interaction, *F*(2, 93) = 3.198, BH-FDR-adjusted *p* = 0.091, η_p_^2^ = 0.064. R-SAC therefore did not show a group-specific interaction after correction. The principal neural results were training-specific reductions in R-M1 and L-FPA activation alongside improved spatial-memory-task performance, a pattern consistent with more economical task-related recruitment within the prefrontal-sensorimotor system.

**Figure 4:**
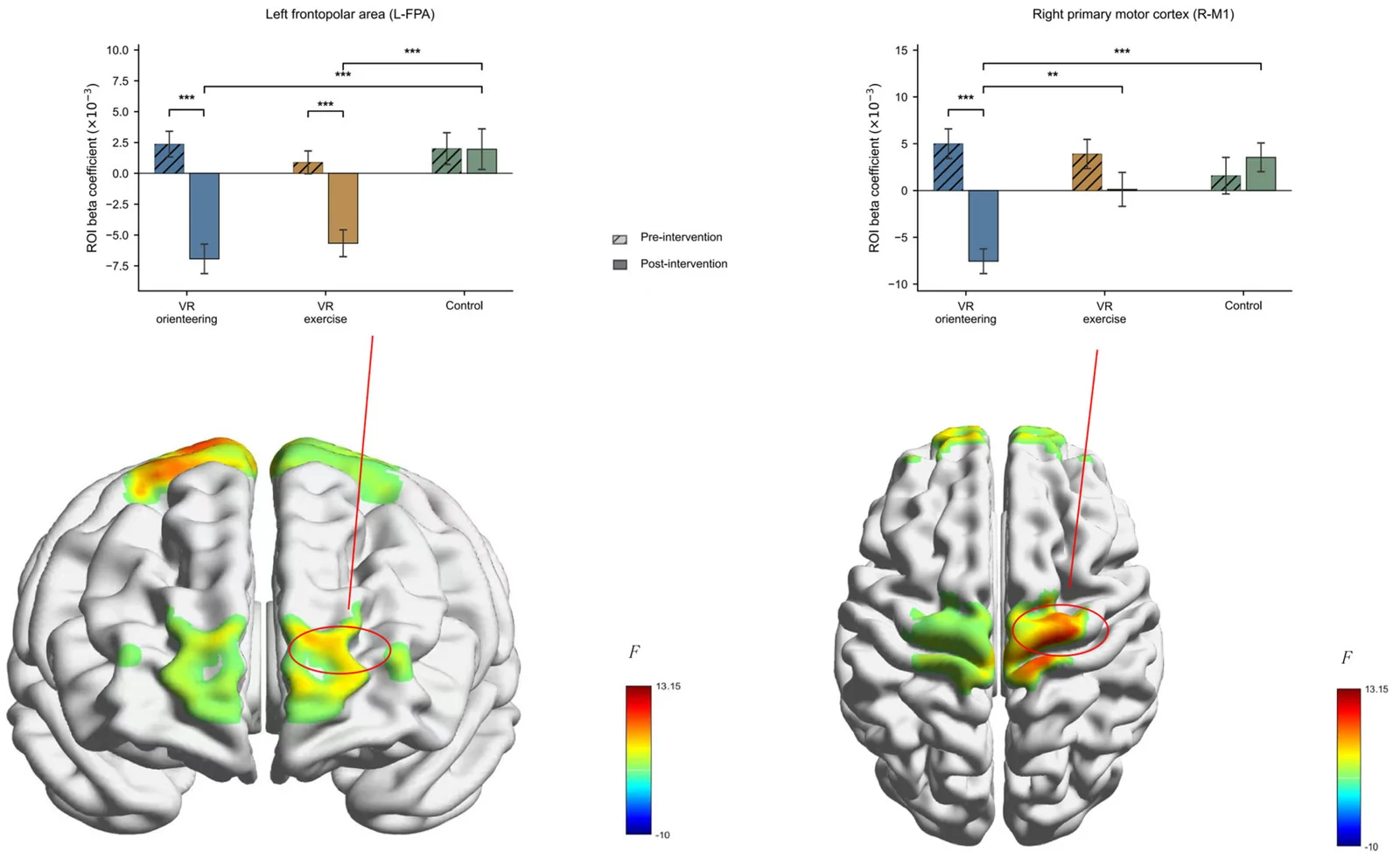
Group × Time interactions in key ROI activation before and after intervention. The surface maps show group-level F statistics and are not single-subject example images; the bar plots show group means ± *SEM*. Color bars are labeled F. Omnibus *p* values are BH-FDR-adjusted across eight ROIs; pairwise *p* values are Bonferroni-adjusted within each ROI. ** *p* < 0.01, *** *p* < 0.001.

**Figure 5:**
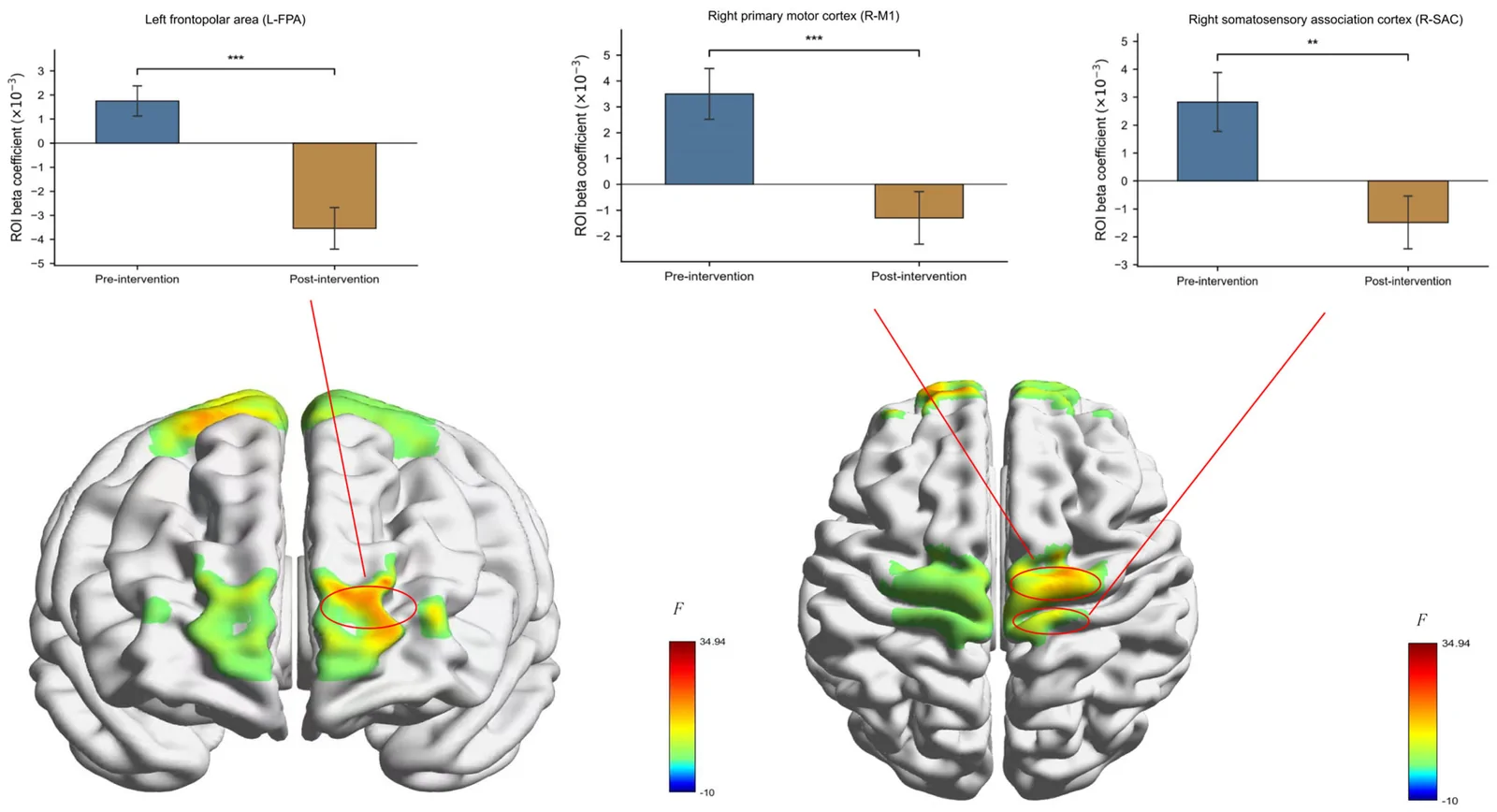
Time main effects on activation in key ROIs. The surface maps show group-level F statistics and are not single-subject example images; the bar plots show group means ± *SEM* (marginal means collapsed across groups). Color bars are labeled F. Omnibus *p* values are BH-FDR-adjusted across eight ROIs; pairwise *p* values are Bonferroni-adjusted within each ROI. ** *p* < 0.01, *** *p* < 0.001.

**Figure 6:**
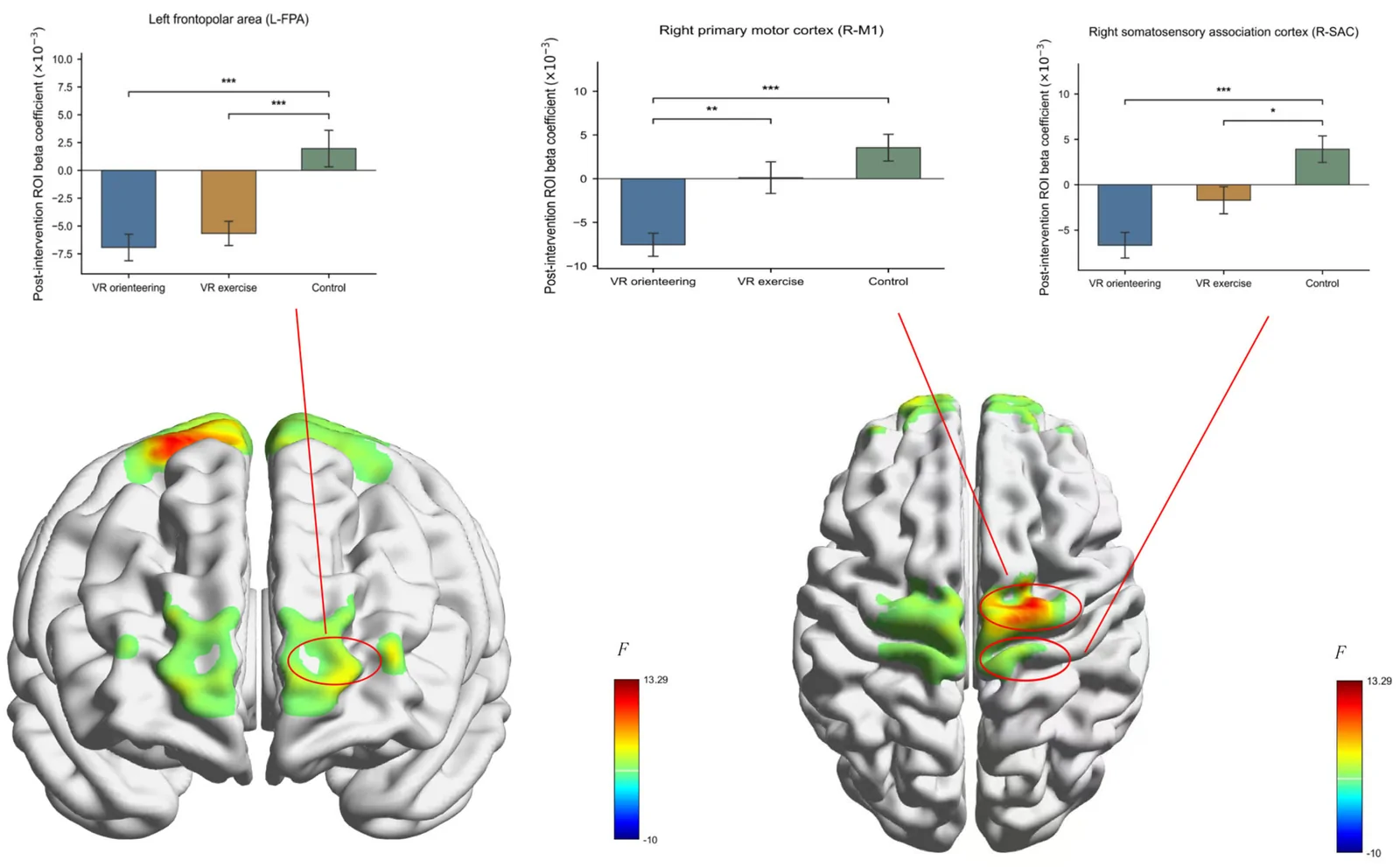
Post-intervention group differences in activation of key ROIs. The surface maps show group-level F statistics and are not single-subject example images; the bar plots show group means ± *SEM* (*n* = 32 per group). Color bars are labeled F. Omnibus *p* values are BH-FDR-adjusted across eight post-intervention ROI-level one-way ANOVAs; pairwise *p* values are Bonferroni-adjusted within each ROI. * *p* < 0.05, ** *p* < 0.01, *** *p* < 0.001.

Figure 4, Figure 5 and Figure 6 present complementary corrected results: Figure 4 shows the significant Group × Time interactions in R-M1 and L-FPA; Figure 5 shows the significant Time main effects in R-M1, L-FPA, and R-SAC; and Figure 6 shows group differences at the post-intervention assessment, evaluated as a separate family of eight ROI-level one-way ANOVAs. The post-intervention Group effect survived BH-FDR correction in R-M1, *F*(2, 93) = 13.155, BH-FDR-adjusted *p* < 0.001, η_p_^2^ = 0.221; L-FPA, *F*(2, 93) = 13.073, BH-FDR-adjusted *p* < 0.001, *η*_p_^2^ = 0.219; and R-SAC, *F*(2, 93) = 13.265, BH-FDR-adjusted *p* < 0.001, η_p_^2^ = 0.222. In R-M1, activation was lower in the VR orienteering group than in the VR exercise group, *t*(93) = −3.463, Bonferroni-adjusted *p* = 0.002, Hedges’ g = −0.846, and control group, *t*(93) = −5.008, Bonferroni-adjusted *p* < 0.001, Hedges’ g = −1.356; the VR exercise and control groups did not differ, *t*(93) = −1.545, Bonferroni-adjusted *p* = 0.377, Hedges’ g = −0.356. In L-FPA, activation was lower than control in both the VR orienteering group, *t*(93) = −4.725, Bonferroni-adjusted *p* < 0.001, Hedges’ g = −1.081, and VR exercise group, *t*(93) = −4.055, Bonferroni-adjusted *p* < 0.001, Hedges’ g = −0.955; the two VR groups did not differ, *t*(93) = −0.671, Bonferroni-adjusted *p* = 1.000, Hedges’ g = −0.193. In R-SAC, activation was lower in the VR orienteering group than in the control group, *t*(93) = −5.147, Bonferroni-adjusted *p* < 0.001, Hedges’ g = −1.289, and lower in the VR exercise group than in the control group, *t*(93) = −2.735, Bonferroni-adjusted *p* = 0.022, Hedges’ g = −0.666; the two VR groups did not differ after correction, *t*(93) = −2.413, Bonferroni-adjusted *p* = 0.053, Hedges’ g = −0.596. Because the R-SAC Group × Time interaction did not survive correction, its post-intervention group difference should not be interpreted as a training-specific change.

### 3.3. Change-Score Brain–Behavior Association

Pearson correlations were calculated between pre-to-post changes in each participant-level ROI mean and pre-to-post changes in spatial-memory-task accuracy and reaction time for each group (Table 5). All changes were defined as pre-intervention minus post-intervention. BH-FDR correction was applied across the eight ROI correlations separately within each Group × Outcome family. Each correlation included *n* = 32 participants (*df* = 30). The focal test of Hypothesis 3 was the association between L-OFC activation change and accuracy change in the VR orienteering group.

**Figure 7:**
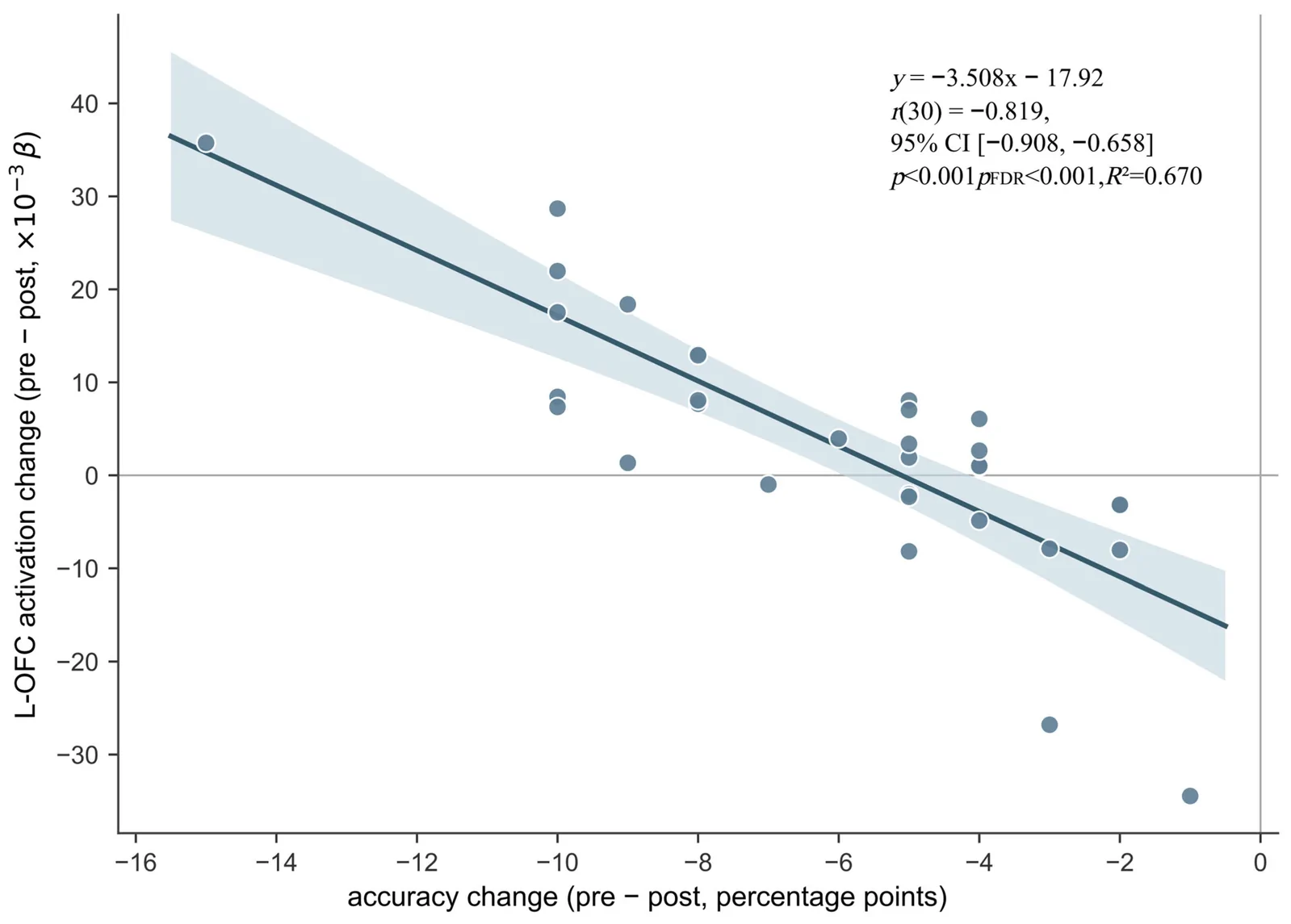
Association between changes in L-OFC activation and spatial-memory-task accuracy in the VR orienteering group. Change scores were calculated as pre-intervention minus post-intervention. Each circle represents one participant. The solid line shows the ordinary least-squares fit, and the light-blue shaded region represents the 95% confidence band for the mean fitted response. L-OFC activation is the participant-level arithmetic mean of channels 9 and 10. Pearson *r*(30) = −0.819, 95% CI [−0.908, −0.658], *p* < 0.001, *R*^2^ = 0.670; BH-FDR-adjusted *p* < 0.001 across the eight ROI correlations in the accuracy-change family; *n* = 32. Leave-one-out Pearson estimates and a Spearman analysis retained the negative direction.

The focal brain-behavior result showed a strong negative relationship between L-OFC activation change and spatial-memory-task accuracy change in the VR orienteering group, Pearson *r*(30) = −0.819, 95% CI [−0.908, −0.658], *p* < 0.001, *R*^2^ = 0.670 (Figure 7). The relationship remained significant after BH-FDR correction across the eight ROI correlations in the VR-orienteering accuracy-change family, adjusted *p* < 0.001. Across the six Group × Outcome families reported in Table 5, no other ROI correlation survived its corresponding eight-ROI correction. Because both changes were calculated as pre-intervention minus post-intervention, the negative coefficient indicates that participants showing larger reductions in L-OFC activation tended to show greater improvements in accuracy. No observation met an exclusion criterion, and no observation was removed. The maximum Cook’s distance was 0.366 (VR05), and leave-one-out Pearson estimates remained negative, ranging from −0.836 to −0.772. A same-data Spearman analysis also retained the negative direction, rho = −0.837, unadjusted *p* < 0.001. Together, the corrected relationship and its stable direction identify L-OFC activation change as a candidate neural correlate of improved spatial-memory performance after VR orienteering.

## 4. Discussion

The findings form a connected sequence: VR orienteering improved laboratory spatial-memory performance, the improvement coincided with reduced recruitment of right M1 and left FPA, and L-OFC activation change tracked the gain in accuracy. The discussion follows this sequence from behavioral improvement to cortical adaptation and the brain-behavior relationship, with laboratory spatial memory as the central outcome.

### 4.1. VR Orienteering and Spatial Memory Task Performance: The Role of Cognitive Participation

Behavioral results showed that 8-week VR orienteering training improved performance on the spatial memory task in participants with operationally defined DTD-like navigational difficulties, and the improvement was greater than that observed in the VR exercise and control groups. In the VR orienteering group, accuracy increased from 73.81% to 79.94%, *t*(31) = 10.895, Bonferroni-adjusted *p* < 0.001, Cohen’s dz = 1.926, and reaction time decreased from 1792.14 ms to 1506.71 ms, *t*(31) = −5.526, Bonferroni-adjusted *p* < 0.001, Cohen’s dz = −0.977. The simultaneous gains in accuracy and speed indicate stronger performance on the spatial memory task without an obvious speed-accuracy trade-off.

The behavioral pattern varied with the cognitive demands of the intervention: the VR orienteering group, which required autonomous navigation, showed the largest improvement, whereas the VR exercise group showed a smaller improvement in accuracy but not reaction time. Both intervention groups outperformed the quiet control group in accuracy. This pattern is consistent with the multi-level framework proposed by Stillman et al. (2020), in which cognitive benefits of exercise may depend partly on the degree of cognitive participation (Yin et al., 2024). VR orienteering requires continuous integration of path, direction, and environmental cues during movement, which may support the encoding and updating of spatial information (Eccles et al., 2002; Stroth et al., 2009). The VR exercise condition provided sensory and motor stimulation without independent spatial planning, so the additional spatial-planning component of orienteering may account for its larger benefit on the laboratory spatial memory task.

From the perspective of embodied-cognition theory, VR orienteering couples body movement with spatial cognition: participants complete map reading, route planning, and decision making while moving in place to drive virtual displacement. This combination provides an embodied-learning context for spatial representation and spatial-memory-related processing (Eccles et al., 2002). Accordingly, spatially guided movement, rather than simple physical activity alone, may be an important ingredient in building the spatial representations recruited by the present task. Direct tests of route memory and real-world navigation will show how far this laboratory benefit extends.

### 4.2. Task-Related Cortical Recruitment and Spatial Memory Improvement: An Interpretation Consistent with Neural Efficiency

After BH-FDR correction across the eight ROIs, the fNIRS results retained intervention-associated reductions in task-related activation in the right primary motor cortex (R-M1) and left frontopolar area (L-FPA) (Miller & Cohen, 2001). Their co-occurrence with improved spatial-memory-task performance gives a coherent signature of more economical task-related cortical recruitment. This post-training pattern differs from the baseline under-recruitment reported in DTD: lower recruitment accompanied by impaired spatial encoding or cognitive-map formation indicates dysfunction, whereas lower activation accompanied by improved performance may indicate a more efficient allocation of task-related resources (Iaria et al., 2009, 2014; Kim et al., 2015; Neubauer & Fink, 2009; Palermo et al., 2014; Poldrack et al., 2005; Rusconi et al., 2021).

The R-M1 finding adds a sensorimotor dimension to the spatial-memory story. Because the task required a binary keypress response, the R-M1 change may reflect a combination of response preparation, sensorimotor integration, embodied spatial representation, and training-related cortical adaptation. Prior motor-learning work has linked practice with changes in M1 activation (Kelly & Garavan, 2005). In the present intervention, repeated spatially guided movement may therefore have reshaped the sensorimotor support recruited during spatial-memory performance.

The corrected L-FPA reduction points to altered demands on high-level cognitive control. FPA (BA10) has been linked to episodic retrieval, multitask processing, and metacognitive monitoring (Miller & Cohen, 2001). Lower L-FPA recruitment may reflect a reduced executive-control demand as participants became more practiced with the spatial-memory task. The R-FPA interaction was not retained after the eight-ROI correction, so the neural pattern centers on the left frontopolar area. The comparison with orienteering athletes (Yin et al., 2024) places this finding within a broader literature on experience-related changes in prefrontal recruitment.

Taken together, the reductions in R-M1 and L-FPA activation form a coherent pattern of lower task-related cortical recruitment alongside better spatial-memory-task performance. The pattern supports a training-related shift toward more economical processing in the accessible prefrontal-sensorimotor system and links the behavioral effect of VR orienteering to a specific cortical signature.

### 4.3. L-OFC Change–Spatial Memory Association

The central brain-behavior finding was the strong relationship between changes in L-OFC activation and spatial-memory-task accuracy. Within the VR orienteering group, participants with larger pre-to-post reductions in L-OFC activation tended to show greater improvements in spatial-memory-task accuracy. This association was strong, *r*(30) = −0.819, and survived BH-FDR correction across the eight ROI tests in the accuracy-change family (adjusted *p* < 0.001). Leave-one-out Pearson estimates and the Spearman coefficient retained the negative direction, indicating that the relationship was stable across observations and correlation estimators. The L-OFC result therefore connects cortical adaptation with the individual variation in spatial-memory improvement produced by VR orienteering.

The functional profile of L-OFC provides a plausible interpretation for this link. L-OFC has been implicated in evaluative processing and perceptual decision monitoring (Fleming et al., 2012; Kringelbach & Rolls, 2004), while neural-efficiency accounts predict that better performance may accompany lower task-related recruitment when processing demands and resource-allocation requirements are reduced (Neubauer & Fink, 2009; Poldrack et al., 2005). Lower L-OFC activation together with higher spatial-memory accuracy may therefore reflect a lighter evaluative or executive-control load during task performance. This interpretation places L-OFC alongside R-M1 and L-FPA in a distributed cortical account of training-related spatial-memory improvement.

Taken together, the multiplicity-corrected L-OFC-accuracy change relationship, its stable direction across secondary analyses, and the supporting functional literature identify L-OFC activation change as a candidate neural correlate of spatial-memory improvement after VR orienteering. Repeated intermediate assessments and larger samples will help trace how this brain-behavior relationship develops across training and how laboratory spatial memory connects with navigation in everyday environments.

### 4.4. Research Limitations and Prospects

Several considerations define the scope of these findings. First, fNIRS captures cortical hemodynamic responses, so the deep hippocampal and retrosplenial components of spatial memory remain outside the present measurement (Pinti et al., 2020). Prior studies have linked navigational experience to hippocampal structure (Maguire et al., 2000) and aerobic training to hippocampal volume and spatial-memory outcomes (Erickson et al., 2011), providing a broader context for the cortical pattern observed here. Second, the task measured delayed visuospatial maintenance, comparison, recognition, and decision making, providing a focused laboratory measure of spatial memory. Its relationship to route memory, route learning, cognitive-map construction, navigation ability, and real-world navigational performance should be examined with dedicated outcomes. Object-based spatial ability has been associated with virtual navigation performance (Garg et al., 2024). A within-subject study comparing one real-world and two virtual navigation paradigms found both shared and paradigm-specific variance, supporting the use of reliable, multi-paradigm assessment and caution against treating virtual and real-world navigation measures as interchangeable (Lader et al., 2025). Visuospatial working memory also plays an important role in indoor wayfinding and direction giving (Hund, 2016), while age differences have been observed in a virtual-environment navigation task (Moffat et al., 2001). Together, these findings suggest overlap, but not equivalence, between component visuospatial abilities and navigation outcomes, and motivate direct tests of route learning and real-world transfer. Third, the post-test session, including the spatial memory task and concurrent task-related fNIRS acquisition, was conducted 48 h after the final scheduled session; no outcome assessment was performed immediately after training. Longer delayed follow-ups will be valuable for charting the persistence of the training-related pattern. Fourth, the change-score analysis captures the relationship between pre-to-post changes and may be influenced by measurement error or regression to the mean; longitudinal designs with repeated assessments can clarify the temporal dynamics. Finally, participants were college students with operationally defined DTD-like navigational difficulties rather than clinically diagnosed DTD patients, so extension to clinical populations or other age groups requires further study.

## 5. Conclusions

Through an 8-week randomized controlled experiment, this study showed that VR orienteering improved spatial-memory accuracy and reaction time in college students with operationally defined DTD-like navigational difficulties. The behavioral improvement was accompanied by training-specific reductions in task-related activation in R-M1 and L-FPA after BH-FDR correction across eight ROIs, linking better spatial-memory performance with more economical recruitment of accessible prefrontal-sensorimotor cortex. Within the VR orienteering group, greater reductions in L-OFC activation were associated with greater improvements in spatial-memory accuracy, and this relationship survived eight-ROI BH-FDR correction. Together, the findings identify a coordinated behavioral-cortical pattern through which VR orienteering may strengthen spatial-memory-related processing and position L-OFC activation change as a candidate neural correlate of individual improvement. The laboratory task captures a core spatial-memory process relevant to navigation; dedicated route and real-world measures will be important for mapping the full extent of this benefit.

## Figures and Tables

**Table 1:** Basic Information of Participants.

Group	VR Orienteering Group	VR Exercise Group	Control Group	*F*	*p*
Gender (Male/Female) (*n*)	16/16	16/16	16/16	—	—
Age (Years)	18.84 ± 0.81	19.06 ± 0.76	18.84 ± 0.68	0.91	0.41
BMI (kg/m^2^)	21.97 ± 1.64	21.27 ± 1.63	21.37 ± 1.72	1.63	0.20
Resting Heart Rate (BPM)	66.50 ± 5.64	68.44 ± 5.05	65.69 ± 5.72	2.13	0.13

Note: BMI = Body Mass Index; BPM = Beats Per Minute. There were no statistically significant differences among the three groups in all variables (*p* > 0.05).

**Table 2:** Distribution table of brain area pathways.

Brain Region	Channel
Left Frontal Pole Area (L-FPA)	Channel 2, 3, 6, 7
Right Frontal Pole Area (R-FPA)	Channel 1, 2, 4, 5
Left Orbitofrontal Cortex (L-OFC)	Channel 9, 10
Right Orbitofrontal Cortex (R-OFC)	Channel 8, 9
Left Somatosensory Association Cortex (L-SAC)	Channel 16, 19, 20
Right Somatosensory Association Cortex (R-SAC)	Channel 15, 18, 19
Left Primary Motor Cortex (L-M1)	Channel 12, 13, 17
Right Primary Motor Cortex (R-M1)	Channel 11, 12, 14

Note: Some channels (such as channel 9) are located at the junction of brain functional areas. According to their spatial probability registration results, they are included in adjacent regions of interest for analysis, which is a common practice in fNIRS research to effectively capture the activation signals of boundary regions.

**Table 3:** Behavioral results and mixed-design ANOVAs before and after intervention (*M* ± *SD*).

	VR Orienteering Group (*n* = 32)		VR Exercise Group (*n* = 32)		Control Group (*n* = 32)		Group	Time	G × T
	Pre	Post	Pre	Post	Pre	Post	*F*; *p*; η_p_^2^	*F*; *p*; η_p_^2^	*F*; *p*; η_p_^2^
ACC (%)	73.81 ± 2.81	79.94 ± 2.73	73.81 ± 2.78	76.66 ± 2.42	74.00 ± 2.55	74.19 ± 2.42	13.008 <0.001 0.219	107.925 <0.001 0.537	34.163 <0.001 0.424
RT (ms)	1792.14 ± 323.70	1506.71 ± 304.67	1654.97 ± 323.97	1762.94 ± 271.65	1805.49 ± 321.17	1823.31 ± 299.60	2.883 0.061 0.058	3.907 0.051 0.040	19.534 <0.001 0.296

Note. The final three columns report *F*, *p*, and partial eta squared (η_p_^2^), in that order. Degrees of freedom were (2, 93) for Group and Group × Time, and (1, 93) for Time.

**Table 4:** Descriptive statistics and BH-FDR-corrected mixed-design ANOVAs for ROI beta coefficients (*M* ± *SD*).

	VR Orienteering Group (*n* = 32)		VR Exercise Group (*n* = 32)		Control Group (*n* = 32)		Time	Group	G × T
	Pre (10^−3^ × Beta)	Post (10^−3^ × Beta)	Pre (10^−3^ × Beta)	Post (10^−3^ × Beta)	Pre (10^−3^ × Beta)	Post (10^−3^ × Beta)	*F*; *p*FDR; η_p_^2^	*F*; *p*FDR; η_p_^2^	*F*; *p*FDR; η_p_^2^
L-M1	3.40 ± 10.73	−3.00 ± 8.43	1.76 ± 8.45	−0.08 ± 9.48	−0.58 ± 12.70	1.66 ± 10.03	2.347 0.147 0.025	0.055 0.981 0.001	3.644 0.080 0.073
R-M1	5.00 ± 8.97	−7.56 ± 7.46	3.91 ± 8.80	0.12 ± 10.25	1.58 ± 11.04	3.55 ± 8.68	14.739 <0.001 0.137	2.846 0.168 0.058	11.435 <0.001 0.197
L-FPA	2.36 ± 5.89	−6.93 ± 6.75	0.89 ± 5.23	−5.67 ± 6.15	2.01 ± 7.27	1.96 ± 9.31	32.813 <0.001 0.261	7.392 0.004 0.137	8.791 0.001 0.159
R-FPA	1.03 ± 9.68	−2.59 ± 5.89	1.47 ± 6.41	−1.01 ± 7.72	1.66 ± 8.46	1.45 ± 10.85	3.331 0.114 0.035	1.152 0.622 0.024	0.755 0.540 0.016
L-OFC	2.81 ± 10.84	−0.75 ± 9.45	3.08 ± 7.59	−2.29 ± 10.85	2.06 ± 11.97	2.59 ± 10.70	4.497 0.073 0.046	0.478 0.829 0.010	1.755 0.238 0.036
R-OFC	3.17 ± 11.75	−1.25 ± 8.96	4.72 ± 7.72	1.55 ± 7.85	2.34 ± 9.60	3.84 ± 9.23	2.883 0.124 0.030	0.955 0.622 0.020	2.270 0.174 0.047
L-SAC	3.36 ± 18.13	0.95 ± 8.38	3.67 ± 8.85	1.42 ± 9.69	1.75 ± 11.12	2.77 ± 7.84	0.613 0.436 0.007	0.019 0.981 <0.001	0.520 0.596 0.011
R-SAC	2.31 ± 9.75	−6.67 ± 7.99	1.85 ± 12.31	−1.71 ± 8.45	4.32 ± 8.77	3.92 ± 8.25	9.487 0.007 0.093	8.013 0.004 0.147	3.198 0.091 0.064

Note. Values are participant-level ROI means (*M* ± *SD*). The final three columns report *F*, BH-FDR-adjusted *p*, and partial eta squared (η_p_^2^), in that order. Degrees of freedom were (2, 93) for Group and Group × Time, and (1, 93) for Time. FDR adjustment was applied separately to each effect across the eight ROIs.

**Table 5:** Pearson correlations between ROI activation changes and spatial-memory-task outcome changes by group.

ROI	Group	Accuracy Change	Reaction-Time Change
L-FPA	VR Orienteering	*r* = −0.027; *p* = 0.882; *p*FDR = 0.924	*r* = −0.197; *p* = 0.279; *p*FDR = 0.653
	VR Exercise	*r* = 0.174; *p* = 0.341; *p*FDR = 0.535	*r* = −0.273; *p* = 0.131; *p*FDR = 0.262
	Control	*r* = 0.049; *p* = 0.790; *p*FDR = 0.903	*r* = 0.281; *p* = 0.120; *p*FDR = 0.671
R-FPA	VR Orienteering	*r* = −0.091; *p* = 0.621; *p*FDR = 0.828	*r* = 0.016; *p* = 0.932; *p*FDR = 0.932
	VR Exercise	*r* = 0.048; *p* = 0.793; *p*FDR = 0.793	*r* = −0.130; *p* = 0.477; *p*FDR = 0.636
	Control	*r* = 0.111; *p* = 0.545; *p*FDR = 0.857	*r* = 0.100; *p* = 0.587; *p*FDR = 0.671
L-OFC	VR Orienteering	*r* = −0.819; *p* < 0.001; *p*FDR < 0.001	*r* = 0.158; *p* = 0.387; *p*FDR = 0.653
	VR Exercise	*r* = 0.247; *p* = 0.173; *p*FDR = 0.535	*r* = 0.000; *p* = 0.999; *p*FDR = 0.999
	Control	*r* = −0.022; *p* = 0.906; *p*FDR = 0.906	*r* = 0.211; *p* = 0.247; *p*FDR = 0.671
R-OFC	VR Orienteering	*r* = −0.391; *p* = 0.027; *p*FDR = 0.107	*r* = −0.151; *p* = 0.408; *p*FDR = 0.653
	VR Exercise	*r* = 0.118; *p* = 0.521; *p*FDR = 0.596	*r* = −0.056; *p* = 0.760; *p*FDR = 0.868
	Control	*r* = −0.349; *p* = 0.050; *p*FDR = 0.402	*r* = 0.121; *p* = 0.509; *p*FDR = 0.671
L-SAC	VR Orienteering	*r* = 0.018; *p* = 0.924; *p*FDR = 0.924	*r* = 0.033; *p* = 0.858; *p*FDR = 0.932
	VR Exercise	*r* = 0.163; *p* = 0.373; *p*FDR = 0.535	*r* = −0.431; *p* = 0.014; *p*FDR = 0.055
	Control	*r* = −0.085; *p* = 0.642; *p*FDR = 0.857	*r* = 0.138; *p* = 0.452; *p*FDR = 0.671
R-SAC	VR Orienteering	*r* = 0.101; *p* = 0.584; *p*FDR = 0.828	*r* = −0.288; *p* = 0.110; *p*FDR = 0.653
	VR Exercise	*r* = −0.157; *p* = 0.391; *p*FDR = 0.535	*r* = 0.243; *p* = 0.180; *p*FDR = 0.288
	Control	*r* = −0.124; *p* = 0.499; *p*FDR = 0.857	*r* = 0.106; *p* = 0.562; *p*FDR = 0.671
L-M1	VR Orienteering	*r* = −0.143; *p* = 0.435; *p*FDR = 0.828	*r* = 0.222; *p* = 0.222; *p*FDR = 0.653
	VR Exercise	*r* = 0.327; *p* = 0.068; *p*FDR = 0.535	*r* = −0.457; *p* = 0.009; *p*FDR = 0.055
	Control	*r* = −0.267; *p* = 0.140; *p*FDR = 0.558	*r* = 0.144; *p* = 0.432; *p*FDR = 0.671
R-M1	VR Orienteering	*r* = −0.160; *p* = 0.381; *p*FDR = 0.828	*r* = −0.109; *p* = 0.553; *p*FDR = 0.737
	VR Exercise	*r* = 0.154; *p* = 0.401; *p*FDR = 0.535	*r* = −0.295; *p* = 0.101; *p*FDR = 0.262
	Control	*r* = −0.092; *p* = 0.616; *p*FDR = 0.857	*r* = 0.073; *p* = 0.692; *p*FDR = 0.692

Note. Change scores were calculated as pre-intervention minus post-intervention. Each cell reports Pearson r, the raw *p* value, and the BH-FDR-adjusted *p* value. Each correlation included *n* = 32 and *df* = 30. BH-FDR correction was applied across the eight ROIs separately within each Group × Outcome family. ROI means used the channels listed in Table 2. Pearson r is the effect size; *R*^2^ is additionally reported in the text for the focal L-OFC-accuracy association.

## Data Availability

The data supporting the findings of this study are available from the corresponding author upon reasonable request.
